# Search, identification, and curation of cell and gene therapy product regulations using augmented intelligent systems

**DOI:** 10.3389/fmed.2023.1072767

**Published:** 2023-03-06

**Authors:** William Schaut, Akash Shrivastav, Srikanth Ramakrishnan, Robert Bowden

**Affiliations:** ^1^Cell Collection, CAR-T Advanced Therapeutics Supply Chain, Janssen Pharmaceutical, Inc., Horsham, PA, United States; ^2^Intelligent Automation and Analytics, Research and Development Business Technology, Janssen Pharmaceutical, Inc., Raritan, NJ, United States

**Keywords:** augmented intelligence, machine learning, CAR-T, regulations, automated systematic search, regulatory documents

## Abstract

**Background:**

Manually keeping up-to-date with regulations such as directives, guidance, laws, and ordinances related to cell and gene therapy is a labor-intensive process. We used machine learning (ML) algorithms to create an augmented intelligent system to optimize systematic screening of global regulations to improve efficiency and reduce overall labor and missed regulations.

**Methods:**

Combining Boolean logic and artificial intelligence (i.e., augmented intelligence) for the search process, ML algorithms were used to identify and suggest relevant cell and gene therapy regulations. Suggested regulations were delivered to a landing page for further subject matter expert (SME) tagging of words/phrases to provide system relevance on functional words. Ongoing learning from the repository regulations continued to increase system reliability and performance. The automated ability to train and retrain the system allows for continued refinement and improvement of system accuracy. Automated daily searches for applicable regulations in global databases provide ongoing opportunities to update the repository.

**Results:**

Compared to manual searching, which required 3–4 SMEs to review ~115 regulations, the current system performance, with continuous system learning, requires 1 full-time equivalent to process approximately 9,000 regulations/day. Currently, system performance has 86% overall accuracy, a recommend recall of 87%, and a reject recall of 84%. A conservative search strategy is intentionally used to permit SMEs to assess low-recommended regulations in order to prevent missing any applicable regulations.

**Conclusion:**

Compared to manual searches, our custom automated search system greatly improves the management of cell and gene therapy regulations and is efficient, cost effective, and accurate.

## 1. Introduction

The manufacture of cell and gene therapeutic products, including chimeric antigen receptor T cell (i.e., CAR-T) therapy products, is regulated by both Good Tissue Practice and Good Manufacturing Practice regulations in the United States. In other countries, applicable regulations may be identified as laws, ordinances, and guidelines, and each country uses their individual regulatory framework to create and publish these requirements (1). Rapid advances in cell and gene therapy products led the United States Food and Drug Administration to provide guidance documents describing its regulatory framework to support the development of new cell and gene therapies that are safe for patients (2–6).

One of the challenges with any supply chain partner or drug product manufacturer is to remain current with new and revised regulatory information. However, cell and gene therapy regulations are not uniformly identified and understood across the pharmaceutical supply chain (7). As our cell and gene therapy program expands globally, there is a need to learn regulations for new countries (8). Given that many suppliers and manufacturers are part of a multi-national supply chain, keeping up-to-date with worldwide regulations becomes more difficult with each additional country. The regulations lack standardization with respect to terminology, format, and requirements across global regulatory authorities. In some instances, format within a single country can be variable, and different terms can be used for the same requirements. The diversity of the types of regulations and the lack of standardized regulatory frameworks for cell and gene therapy products create a challenge to search for and identify applicable information (9).

Our current process to identify relevant regulations, defined in this paper as regulatory documents associated with cell and gene therapy products such as directives, guidance, laws, and ordinances, has historically been a labor-intensive, costly (with respect to number of full-time equivalents), and time-consuming endeavor (10) that involves manually searching, identifying, reviewing, curating, and highlighting applicable regulatory requirements, which can also be error-prone due to the human element. To remain up-to-date with regulations, the manual process must be repeated periodically, looking back to the previous evaluation to ensure any updated regulations are recovered and brought into the repository. The frequency of repository management (i.e., the addition of new regulations and removal of outdated regulations) determines the level of acceptable risk. Frequent manual searches, while labor intensive, lower the risk of not complying with a recently approved regulation, whereas infrequent searches reduce resource demand while increasing the risk of regulatory noncompliance.

Before recent advances in artificial intelligence (AI), automating our search process has been extremely difficult due to differences in the regulations. However, augmented intelligence (AuI), which combines Boolean logic and AI (11), proposes to reduce the massive amount of work required by human involvement to perform systematic reviews (12–14). The use of machine learning (ML) algorithms, a subset of AI (15), has previously been demonstrated to assist with categorized document selection in systematic literature reviews, and its use, along with text mining, can reduce manual effort and reliance on humans and increase the efficiency of systematic literature reviews (10, 16). For example, O’Mara-Eves et al. (17) suggested that a workload reduction of 30–70% might be possible with semi-automation, and Cohen et al. (12) reported that a ≥50% reduction of the effort required to review articles manually would provide substantial benefit.

In general, essential tasks for systematic searches include identification of relevant documents, followed by classification and extraction of pertinent information (18). Development of an automated information extraction system to assist with natural language processing (NLP) and text mining can improve the efficiency of this complex process, especially when increasing numbers of documents need to be searched (18). The steps involved in optimizing an ML application consist of preparing and uploading data to be used, model creation, and feature selection to improve the model, followed by an iterative process of building, training, optimizing, validating, and selecting a specific ML algorithm suitable to the task at hand (19). While automation can simplify the search and review process, it still requires human interaction to function since AuI requires a constant flow of data, whereas humans are dynamic and can respond quickly when needed (20). The purpose of this paper is to provide a real-world example of using AuI to develop a customized automatic systematic search system that reduces, but does not eliminate, human intervention to monitor worldwide regulations associated with cell and gene therapy.

## 2. Method

Our AuI system uses a Natural Embeddings for Language Processing (NELP)-Janssen Research and Development Intelligent Automation and Analytics-developed AI-NLP-enabled Intelligent Automation platform to search regulations related to the manufacture of CAR-T therapy products. The goals of developing this custom automated search system were to increase delivery of recently published relevant regulations, increase the quality of search results, instill confidence that relevant regulations would not be missed, and achieve these goals faster and more economically than the current manual process.

### 2.1. Technology

The repository containing cell and gene therapy regulations is powered by NELP which leverages Ensemble ML methods and offers a “recommendation engine,” proprietary explainable AI, and intelligent “duplicate” detection models. The system uses proprietary pre-screening technology to enhance imaging of relevant content in the repository by focusing on key words and phrases that identify a regulation as being in-scope of the intended repository. The automated ability to train and retrain the system allows for continued refinement and improvement of system accuracy, which is used by the system searches in the regulation database to further identify regulations that may be in-scope; we used the Cortellis regulatory intelligence global database (21) since it was accessible from our Regulatory Affairs team.

#### 2.1.1. Natural language processing and its application to the CAR-T domain

Cohen (22) defines NLP as the study of computer programs that take natural or human language as input. Several articles have been published describing the use of NLP in the biomedical domain, including a description of general applications (23) and a description of the use of mining Electronic Health Records and linking genomic and clinical data for personalized care (24).

The use of NLP for precision medicine is relatively less researched. Espinal-Enríquez et al. (25) provided a detailed study of software tools, as well as biological databases, ontologies, and applicable standards. Use of NLP in the field of CAR-T therapy is extremely rare. As of January 2023, we found no references in the Web of Science engine while the Scopus search engine produced one relevant, extremely recent research paper by Zhang et al. (26). We therefore believe that our application of advanced NLP to mine and analyze CAR-T government regulations is the first of its kind in the pharmaceutical industry.

#### 2.1.2. Design and elements of the architecture

The primary design elements considered were (1) the primary repository of regulations documents, Cortellis, which is an industry-standard tool for obtaining regulatory and compliance documents for life sciences across the globe, and (2) the AI-based intelligence automation which consists of the following key components that collectively form the GOLD Standard system: (a) Document pre-processing (translation for non-English regulations and processing unstructured text in PDF documents), (b) NLP-based search engine, (c) Recommendation engine, (d) Active Learning, (e) Document similarity, and (f) Summarization.

##### 2.1.2.1. Document pre-processing

As these regulations are mainly in PDF format, we apply standard computer visions to parse the PDF content and then use “Google translations” service to translate non-English documents to English.

##### 2.1.2.2. Implementing the natural language search

Natural language-based searches have become a common requirement of users in today’s time. Robust, high-speed, at-scale searches were key objectives. We implemented this by leveraging Elasticsearch (Elasticsearch B.V., Mountain View, CA, United States), as a foundation. Then, we implemented vector-based search (that uses Lucene’s vector fields) and search based on approximate nearest neighbor in turn using Hierarchical Navigable Small World search.

##### 2.1.2.3. Implementing the recommendation engine

Recommending regulation documents for review is the core feature of this application. Unstructured text and no initial training examples were the biggest challenges.

###### 2.1.2.3.1. Design and evaluation

The recommended to non-recommended documents ratio was 5:95. The “Decision Threshold” was set for lower False Negatives versus being accommodating toward False Positives. Consequently, the classification metric Recall (1) was more important.


(1) $$Recall=\frac{TP}{\left(TP+FN\right)}$$

Where TP = True Positives, FN = False Negatives.

Evaluation was performed “on-line” by users, feeding back evaluated documents into the Active Learning loop, which is described in Section 2.1.2.4.

###### 2.1.2.3.2. Modeling

Identifying a relevant document, among thousands, at a signal-to-noise ratio of 5:95, is a complex task. We evaluated several algorithms, including Logistic Regression, XGBoost, and Support Vector Machines, for the recommendation engine, selecting Support Vector Machines for its robust performance. For the document “Summarization” task, linguistic modeling was evaluated using attention and transformer models such as BERT, GPT-2, and XLNet, and BERT was selected based on performance and algorithm stability across variations. We trained Support Vector Machines with a Radial Bias Function kernel (2). *Document_1_* and *Document_2_* are multi-dimensional vectorized representations of the documents. A very high order of dimensions (0.5 M) must be used to recommend articles. Support Vector Machines pose hyperparameter tuning challenges that were addressed using grid search.


(2) $$K\left(Document_{1},Document_{2}\right)=\exp\left(-\frac{{\left\|Document_{1}-Document_{2}\right\|}^{2}}{2\sigma^{2}}\;\right)$$

##### 2.1.2.4. Active learning

**Figure 1:**
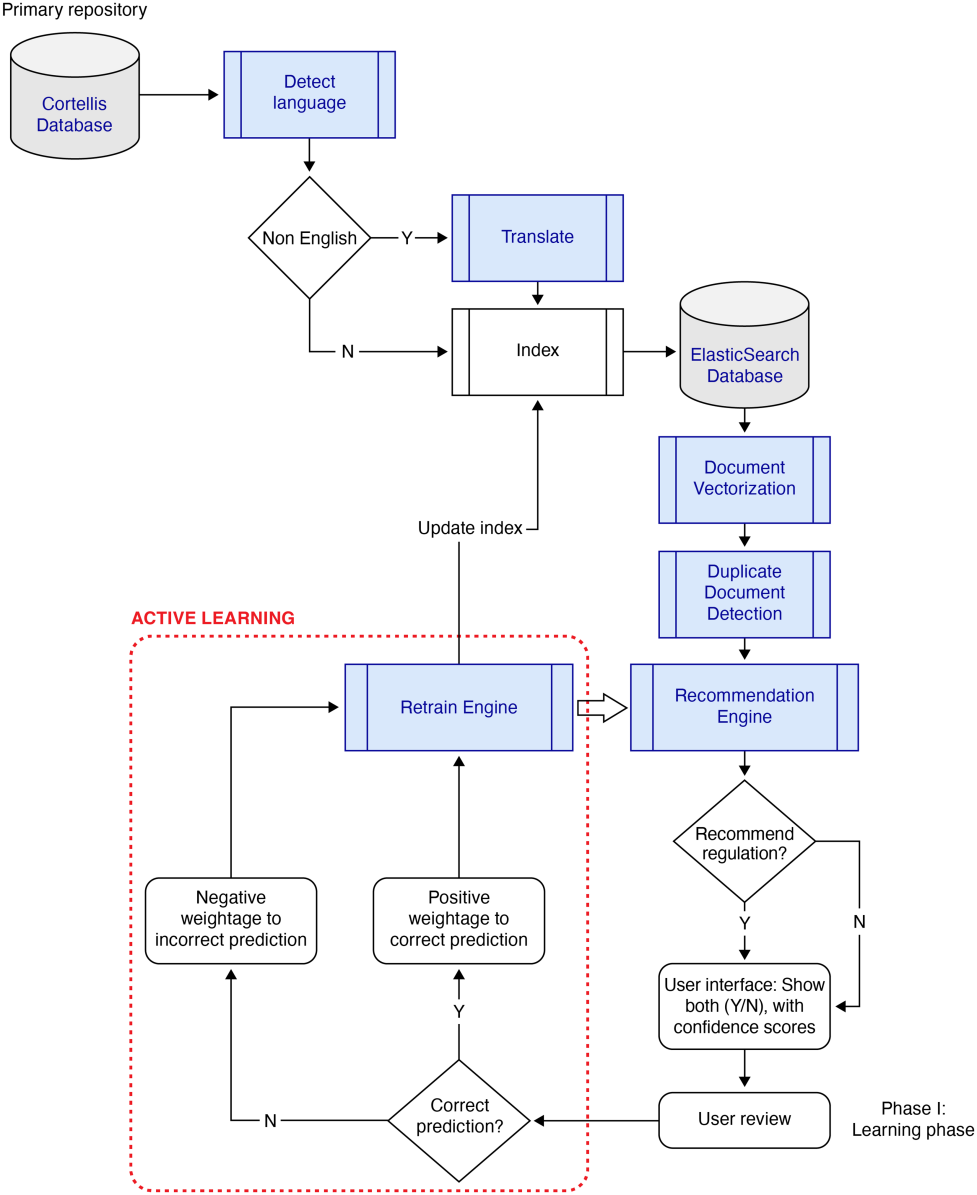
Flow diagram of “Active Learning” for the system. N, no; Y, yes.

To handle low training samples, the system implemented “Active Learning,” shown in Figure 1. As new documents were evaluated using an initially trained model, the predicted documents were presented to the user with a confidence score. Users agreed or disagreed with the prediction. The documents were attached a weight depending on the agreement, thereby reinforcing the decision made by the model. For example, a recommended document that was agreed upon by the user was positively reinforced, while disagreement by the user was negatively penalized by the Active Learning engine. After assessing a pre-defined volume of the training sample, the model was replaced if the evaluation metric was significantly different from the active model in production.

##### 2.1.2.5. Implementing document similarity

Documents create dense vectors. Detecting similar documents with small changes is a challenging task. We used the *k*-nearest neighbor algorithm based on the Hierarchical Navigable Small Worlds algorithm for an efficient match.

##### 2.1.2.6. Implementing generative AI for summarization

Advanced features such as an automatically generated English language summary for both English and non-English regulations and a weekly email digest of newly recommended regulations to a select set of users are available in this application.

The transformer models evaluated for this purpose were Google T5, BERT, and GPT. The ensemble of transformer models is based on a modular architecture, which allows any new technology developments in this area to be plugged in with minimal impact on the overall system.

Natural language processing technology is growing at a rapid pace. Our architecture allows us to adopt new technology easily since we are able to swap the language models with models possessing newer developments. Question-Answer models, interactive agents such as chat-bots (e.g., OpenAI’s ChatGPT), and audio to natural language text to structured query (known as Natural Language Querying) are a few such significant enhancements that can be easily integrated with this architecture to provide a more advanced interface to query the resources in the future phases.

### 2.2. Manual search

During our manual search process, subject matter experts (SMEs) executed a search of Cortellis using a string of Boolean logic terms with specific terminology and spelling, with a country-by-country process, to focus on specific regulations. The applicable regulations identified from the manual searches were then used to provide the initial AuI system learning during the pilot phase.

### 2.3. Pilot phase

**Figure 2:**
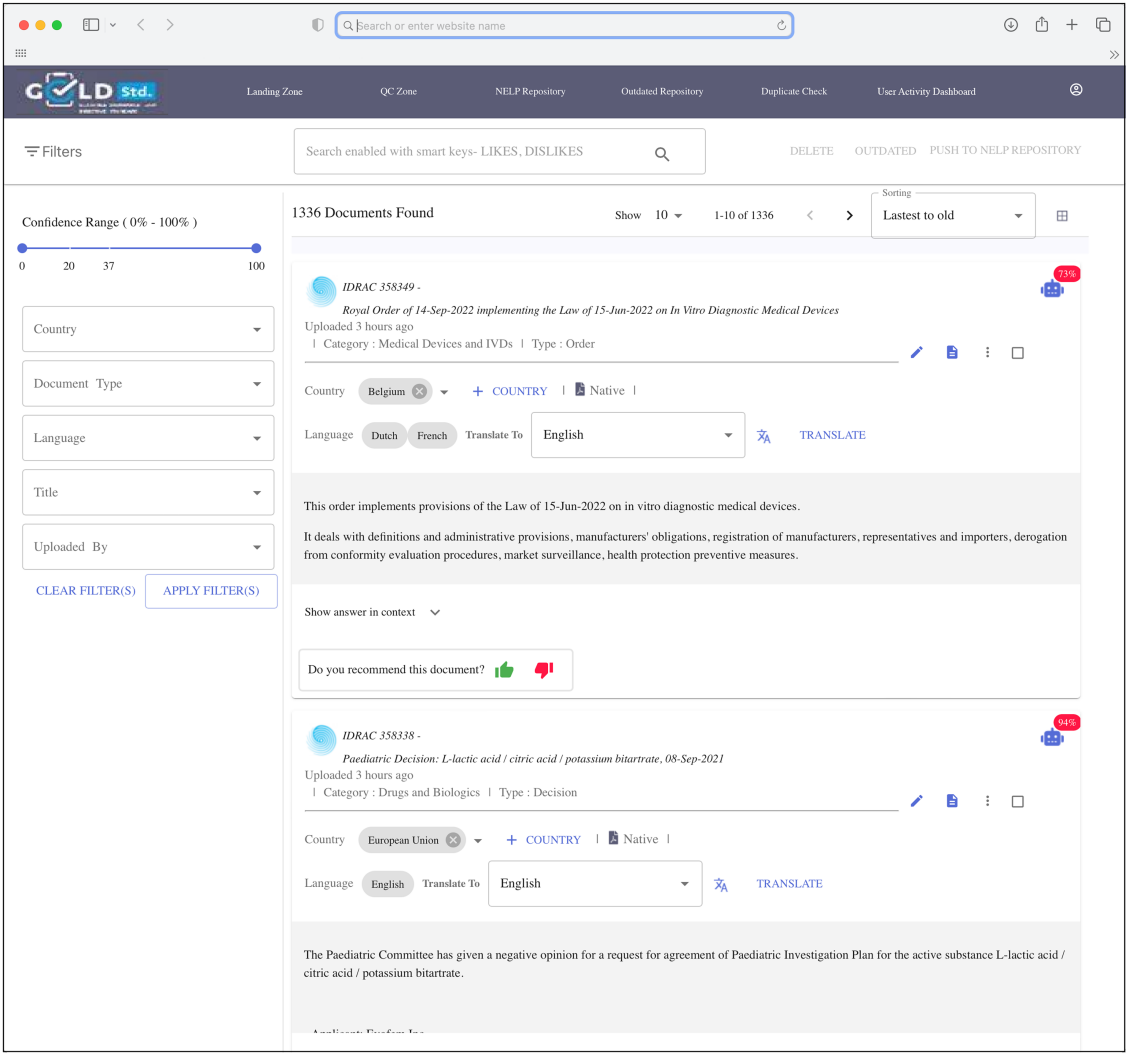
Screen shot of landing page.

The goal of the 3-month pilot phase was to demonstrate the capability of the automated machine search system to discriminate between in-scope and out-of-scope regulations and compare favorably with manual search results. To begin this phase, applicable cell and gene therapy regulations identified in the manual search were pre-selected by SMEs and uploaded into the repository to serve as the initial regulations to build the repository and provide a positive learning environment for the system. An automated daily search was programmed to identify new and updated applicable global regulations from Cortellis and was performed in parallel with the manual searches to check whether the automated search system missed any manually identified regulations. During the annotation step, the regulations were sent to a landing page (Figure 2) for review by SMEs, who specified whether to move each relevant regulation to the repository by annotating as “recommended,” or to delete regulations that were out-of-scope by annotating as “rejected.” Original and new regulations in the repository provided opportunities for ongoing system learning. Rather than applying system learning to the entire regulation (some of which were several hundred pages long), the system could positively and negatively learn on a very small set of single words or phrases consisting of no more than 10 words, thus significantly reducing learning duration and the number of regulations required for learning.

### 2.4. Commercialization phase

Following completion of the pilot phase, the commercialization phase continued to apply system learning to the cell and gene therapy regulation repository. This next phase required building the user experience and optimizing the system to permit simple use and evaluation of applicable regulations, with the goal of streamlining the search process and minimizing search requirements. The system was initially designed to bring all regulations to the landing page and provide an accuracy rating score. Parameters used to assess the AuI system included recommend recall (compared to actually recommended regulations, the number identified by the AuI system as recommended), recommend precision (regulations identified by the AuI system as recommended that are actually recommended), reject recall (compared to actually rejected regulations, the number identified by the AuI system as rejected), and reject precision (regulations identified by the AuI system as rejected that are actually rejected). Data mining in this phase continued until we searched through 20 years of outdated regulations to add to our repository and provide additional system learning opportunities, some of which used different terminology than what is currently used for cell and gene therapy products.

### 2.5. Commercial phase

Once the commercialization phase was complete, the commercial phase is expected to maintain the repository with the most up-to-date regulations from the previous 24 h. To achieve this goal, the system searches the database each day for any new relevant regulations. This phase is ongoing since finding the most current regulations and adding them to the repository is essential to ensure that no important regulations are missed.

### 2.6. Automation process

One key feature of this AuI search system that requires ongoing evaluation is a quality check of the system learning to monitor potential drift from the in-scope regulations. To minimize the system risk, we implemented a second SME quality control system for regulations on the landing page. This quality control system requires input from 2 SMEs to both delete a regulation from the landing page or add one to the repository. Since introduction of non-applicable regulations (either on purpose or by error) could quickly degrade system performance, this 2-step system prevents actions taken by any single individual that could inadvertently add an out-of-scope regulation or delete a regulation that should be added to the repository, which can result in system scope expansion and system performance degradation. The standard for the results evaluation of our automated search system was the daily comparison to the results of manual searches performed by SMEs, over a 7-month period of time.

## 3. Results

**Figure 3:**
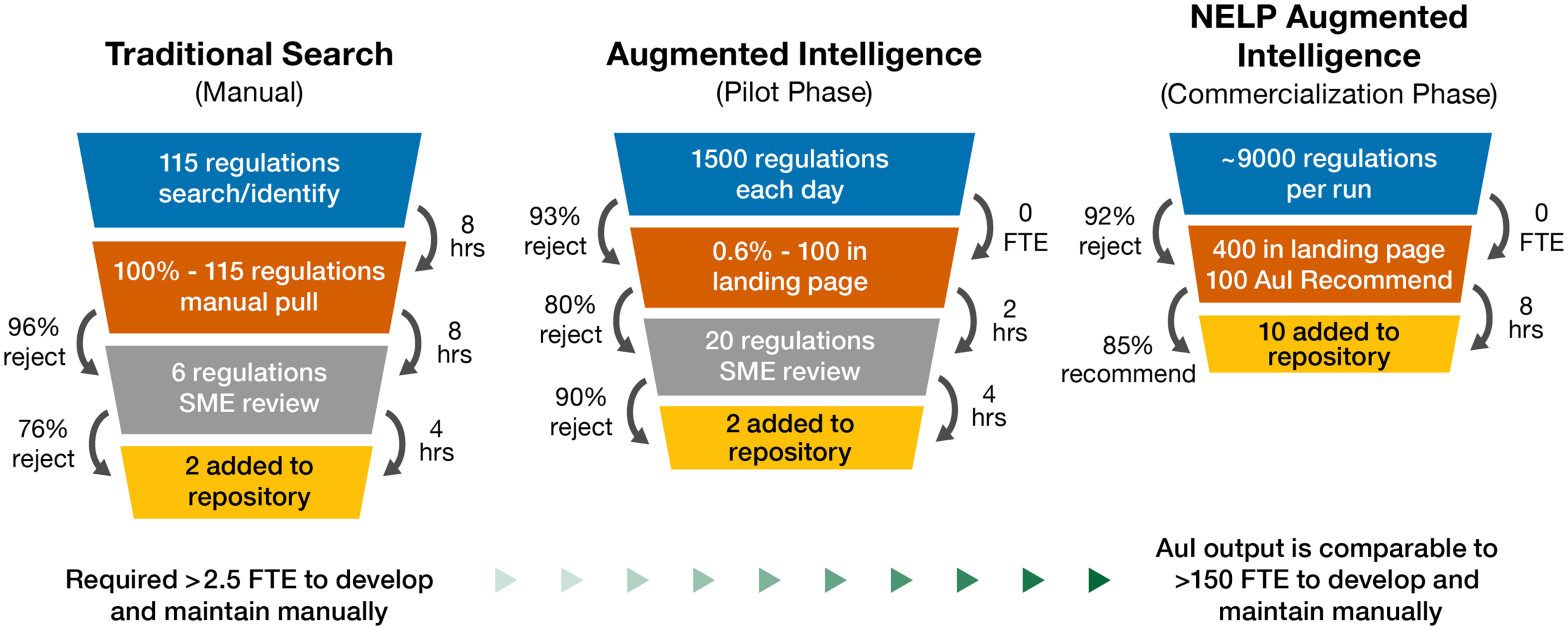
Natural embeddings for language processing (NELP) automation value realization for cell and gene therapy regulations repository: performance outcomes for traditional manual, augmented intelligence, and NELP intelligence search systems. AuI, augmented intelligence; FTE, full-time equivalent; hrs, hours; SME, subject matter expert.

Our novel AuI automated search system was able to predict the best outcomes and identify applicable regulations associated with cell and gene therapy products. The regulatory intelligence database Cortellis posts several thousand regulations each year as observed during our initial searches; however, our search results yielded very few regulations, with even fewer applicable to cell and gene therapy products. During our manual search process, a typical return was approximately 115 regulations. The SMEs validated recovered regulations by reviewing for applicability. Further study and analysis of each country’s regulations often identified 1–3 regulations in-scope with cell and gene therapy products. This manual search process often required 3–4 SMEs (involving quality, technical, regulatory, and legal departments) to identify the country-specific requirements, and ~20 h to review each country. This was a one-time search that needed to be manually repeated to remain current and search different countries (Figure 3). Manually searching for cell and gene therapy regulations across 27 countries, and respective regulatory agencies, yielded 167 regulations (~6 regulations per country) to use to begin the pilot phase.

In the 3-month pilot phase combining Boolean logic and ML to focus system learning, system capability was improved compared to manual searches, such that approximately 1,500 regulations were screened each day, with about 100 arriving at the landing page. A brief overview of the regulation and its title allowed for a quick decision about relevance and eliminated non-applicable regulations, resulting in approximately 20 regulations requiring SME review, and ultimately identifying 1–2 applicable regulations to be uploaded to the repository per day. SME resourcing was then decreased to approximately 1 full-time equivalent to manage the automated searching of 1,500 regulations each day (Figure 3). The pilot phase of the novel AuI search system also identified regulations that were missed by the manual phase since regulations that were not identical to the Boolean logic of the manual searches were able to be identified.

Due to the variability of regulations as well as the smaller number that were specifically relevant to cell and gene therapy, we quickly recognized that system learning would not significantly improve until all applicable regulations were identified due to an imbalanced data set where there was a significant number of non-applicable (>10,000) versus applicable (<1,500) regulations being returned to the landing page. To address this high level of imbalance, the dataset was sampled to make it balanced, such that rejected articles were downsampled (also called under sampled) (16) and made equal to the number of recommended articles. Using Boolean logic, non-applicable regulations routinely being returned by these searches were identified and programmed to be eliminated before arriving at the landing page during the pilot phase. This approach resulted in a gradual increase in the number of applicable regulations while the number of non-applicable regulations decreased, thus increasing the repository data set and improving system functionality. A second action of tagging regulations for words and phrases that would make the regulation inclusive (tagged as “recommended”) or words and phrases that would cause the regulation to be deleted (tagged as “rejected”) also improved the system focus on functional words.

Following successful demonstration of the AuI system capability to identify and build a repository of relevant regulations, we began the commercialization phase with the goal to simplify the human interface, thus improving the user experience. At the beginning of this phase, the AuI system yielded many returns that were not relevant, but as it continued to learn which regulations were applicable and which were not, the returns steadily improved as the system focused more tightly on applicable regulations while rejecting a greater number of out-of-scope regulations. Initially, it took about 12 days to review 1 month of search results because the system was not yet discerning enough to eliminate irrelevant information. As the efficiency and search criteria improved, 2 months of returns could be reviewed every day with fewer regulations reaching the landing page; system recall and accuracy improved over the commercialization phase until the current efficiency was achieved. After 9 months of data mining 20 years of cell and gene therapy regulations, the AuI system ultimately required only about 8 h to process approximately 9,000 regulations each day, with 400 regulations arriving at the landing page, which were quickly reviewed and, by using system recommendations, reduced to ~10 relevant regulations to be added to the repository (Figure 3). Searching older regulations refined the AuI search system and enabled expansion of the system’s capacity to recognize terms associated with relevant regulations across many years.

**Figure 4:**
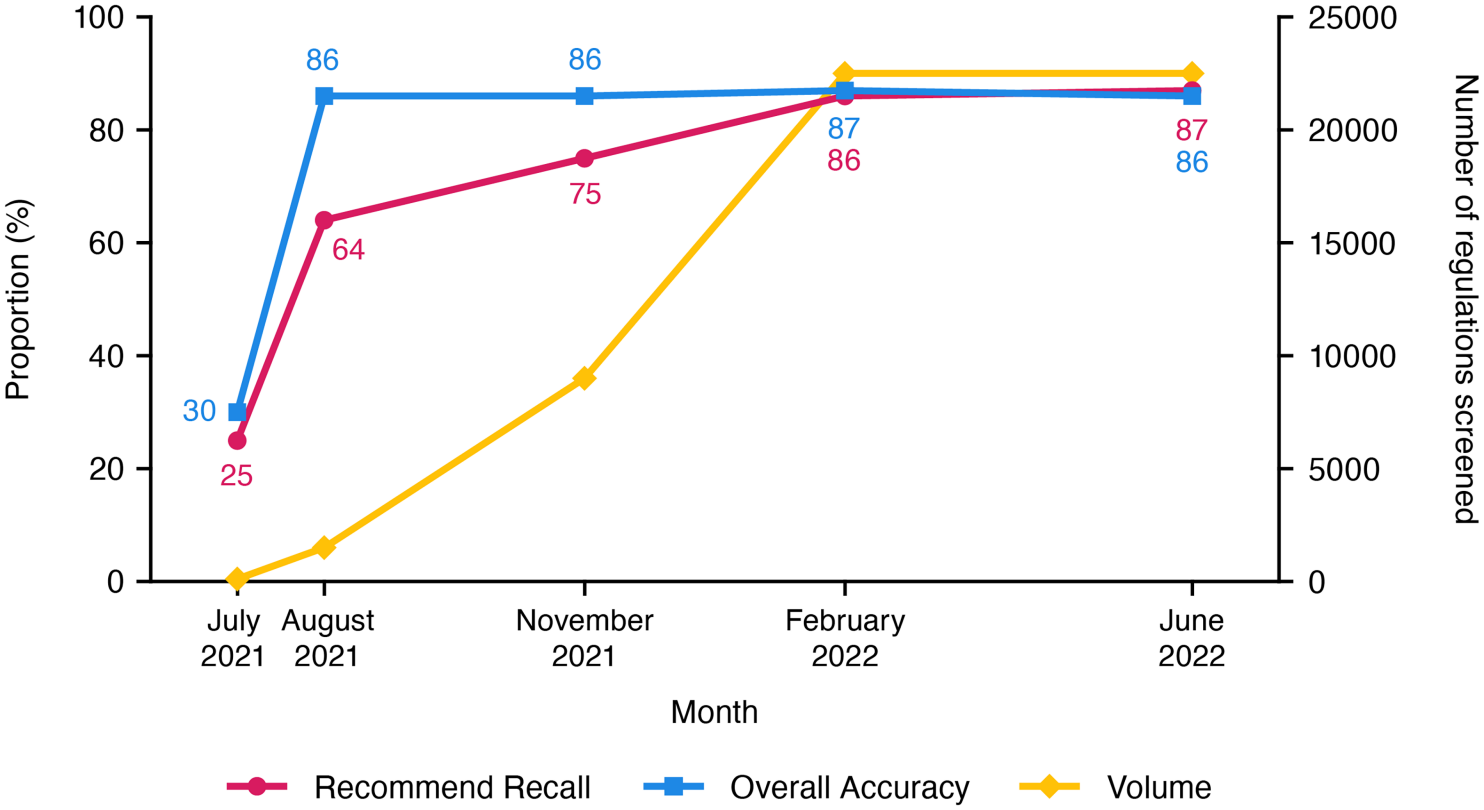
Recommend recall and accuracy values, as well as volume of cell and gene therapy regulations screened, during the development of the customized augmented intelligence search system for cell and gene therapy regulations, through the end of the commercialization phase.

The ongoing commercial phase now performs systematic searches that are limited to regulations uploaded in the past 24 h and takes less than 2 h each day to search for ones relevant to cell and gene therapy (Figure 3). To date, the use of our AuI search system has reduced the manual efforts of regulation mining and curation by 87% (recommend recall), such that the AuI system is correctly identifying recommended regulations 87% of the time (Figure 4), and fewer full-time equivalent resources are needed to execute tasks that the system can now perform. Currently, with a processing time of 3 s per regulation, the system performance has an accuracy of 86%, a recommend precision of 85%, a reject recall of 84%, and a reject precision of 86% (Figure 4), while reviewing approximately 9,000 regulations each day. In total, about 210,000 regulations were mined and 5,000 learning opportunities were provided to teach this novel AuI search system to make critical decisions and increase our confidence that the system is properly identifying relevant regulations.

## 4. Discussion

The novel AuI automated search system described in this paper is able to predict the best outcomes and identify applicable regulations associated with cell and gene therapy products, demonstrating that this technology is beneficial for keeping up-to-date with regulations. Manual searches are limited by the specific Boolean search terms used, such that regulations could be missed simply due to differences in regional spelling or a spelling error. Compared to manual searches, this AuI system provides improved reliability and speed of identifying in-scope regulations with decreased overall cost (including labor, systems, and database) and reduced amount of human intervention required to perform this important process.

The use of manual reviews to train an ML algorithm was previously reported to be approximately 80% accurate at predicting which systematic review articles to include (27). Building our AuI system was initially challenging because it required resources to support comparison of the manual search and 3-month pilot phase results in parallel, as well as to manage the many non-applicable returns during the preliminary stages of learning in the commercialization phase. It was important to keep up with the demand of constantly uploading regulations to maintain the system learning. The benefits provided by the AuI system compared to manual searching became apparent during the pilot phase and persisted through to the commercial phase. Once fully established, performance of the AuI system runs 24 h each day, 7 days a week, providing real-time access to cell and gene therapy regulations, which is a level of productivity that is not sustainable for humans. While not completely eliminating the need for SMEs, the AuI system complements human capability by allowing the SME to focus on activities and decisions that are not well suited to automation. The achieved reduction in manual labor (ranging from 84 to 90%) is consistent with other reported increases in efficiency of systematic literature reviews using ML algorithms and text mining, and reduces the burden on human screeners (12, 13, 16).

Goh (28) supported the use of ML to perform complex searches and reported that “almost perfect” recall for humans correlates with kappa greater than 0.8 where 2 SMEs independently identify the same action for a document. This includes both individuals stating the document should be included or both individuals being in agreement that the document should be rejected. Mismatch and violation of recall occur when the 2 SMEs disagree, such that one SME suggests recommending the document and the second expert recommends rejecting. The automated search system that we developed achieved a recommend recall of 0.87 while also reviewing approximately 9,000 regulations each day.

The wide range of accuracy of different automatic literature screening models is related to the search topic and algorithms used (12, 14). One notable feature of using an AuI search system is the number of learning opportunities and data examples required to increase system performance. We used about 5,000 regulations to teach the system, which provided sufficient learning information about these complex documents; the more regulations used to teach the system, the better the system can be at finding relevant information. In previous published reports, at least 500 opportunities were provided to enhance system learning (29, 30). Jaspers et al. (31) recommended having a human reviewer assign relevance to approximately 1,500 abstracts containing relevant and irrelevant information, which were considered to be 50% of the training data, in order to train an automated systematic search model. Another ML approach that was applied to the systematic review of preclinical animal studies used 5,749 records to train the system and reached a high level of specificity (86%) (32). An AuI model for pharmacovigilance was trained using 20,000 individual case safety reports over a 2-year period, and reached an accuracy score of ≥75%, which was considered the threshold for the AuI system to be considered sufficiently trained and functional (13). The overall accuracy (86%) of our novel AuI search system for curating relevant global regulations with an imbalanced data set is comparable to or an improvement over these reported metrics.

In systematic literature reviews, class imbalances can occur when the number of excluded publications exceeds the number of included citations, and this situation can negatively affect the model performance due to the complications that can arise in training the algorithm with imbalances (16). In addition, previous studies have reported that the savings in workload can sometimes occur at the expense of losing relevant studies (approximately 5%), resulting in a 95% recall (17). Ongoing system checks can be performed to ensure that system accuracy and performance remain high, and that degradation of system performance can be detected before negative consequences occur.

Implementing AuI supports the consistent decision-making required with SMEs by reducing the number of regulations that would otherwise need to be curated manually (13). While our novel AuI system does not entirely replace the human element when searching for relevant regulations, the use of human SMEs can be focused on making critical decisions about the recovered regulations, thus reducing required resources and overall cost, rather than spending hours looking for regulations that may not exist. Although the benefits of ML are widely recognized, the involvement of human experts is still required since algorithms cannot perform well for all possibilities (i.e., No Free Lunch) (19, 33). Popoff et al. (16) also proposed combining ML with human screeners because 100% sensitivity is not guaranteed since not all publications in systematic literature searches can be excluded with a reason.

Production of novel cell and gene therapy products creates many technical as well as regulatory challenges (34–36). Suppliers of starting materials, active pharmaceutical ingredients, and products for cell and gene therapies need to remain diligent in keeping up with new and updated regulations to maintain regulatory compliance, which can be assisted by SMEs routinely performing manual searches or by using AuI to perform most of the work. Since updating regulation repositories with current information and terminology will need to continue as new cell and gene therapy products are discovered, our AuI search system will remain of value to find, sort, translate, and identify specific requirements within very large and complex regulations.

Now that we have established an AuI automated search process for identifying cell and gene therapy regulations, we use it with confidence to efficiently identify new regulations. System performance today operates on the conservative side, bringing low-recommended regulations to the landing page for SME assessment. This strategy was purposely chosen because the risk of missing a regulation is too great, and therefore, we prefer to bring low-recommendation-scored regulations to the landing page rather than prevent those regulations from being assessed by the SME for relevance.

Automatic methods to systematically screen biomedical documents have been researched in only a limited capacity to date (14). We have demonstrated that our custom system for AuI-enabled regulation monitoring vastly improves the proactive management of regulations related to cell and gene therapy compared to manual searches. Although we chose to use Cortellis as the database to search for cell and gene therapy regulations, the technology of our automated search system is readily applicable to other databases (including the open web if security is not a concern) and other search topics, highlighting the versatility of our AuI approach for performing frequent complex searches. Although initial system implementation is costly, the return on the investment is very short in reducing the number of SME resources and the potential cost of lack of compliance caused by failing to identify and implement a regulatory requirement. Future plans for our AuI-enabled regulations repository include assessment of standard operating procedures; ensuring requirements are appropriately reflected in our day-to-day processes associated with cell and gene therapy production; and further expanding the user experience to identify, search, and translate regulations to a chosen language, and summarize multiple regulations.

## Data availability statement

The datasets presented in this article are not readily available because the automated system cannot be shared outside of Johnson & Johnson at this time. Requests to access the datasets should be directed to WS, WSchaut@its.jnj.com.

## Author contributions

WS: contributed design, implementation, data analysis, interpretation, manuscript writing and reviewing, and final approval of manuscript. AS and SR: conceptual design and system build, manuscript reviewing, and final approval of manuscript. RB: conceptual design and study supervision, resourcing, data review and iteration improvement, manuscript writing, and final approval of the manuscript. All authors contributed to the article and approved the submitted version.

## Funding

The authors declare that this study received funding from Janssen Pharmaceutical, Inc. The information contained in this publication belongs to Johnson & Johnson and/or its affiliates. Johnson & Johnson does not make and expressly disclaims: (a) any representation or warranty (express or implied) with respect to the information shown in this publication; and (b) any liability relating to the accuracy or completeness of the information. The views and opinions expressed in this publication are those of the author and do not necessarily reflect the official policy or position of Johnson & Johnson or any of its employees.

## Conflict of interest

All authors are employed by the company Janssen Pharmaceutical, Inc. Employees of the study funder had the following involvement with the study: design, data collection and analysis, interpretation of data, writing and approval of this article, and decision to publish. All authors declare no other competing interests.

## Publisher’s note

All claims expressed in this article are solely those of the authors and do not necessarily represent those of their affiliated organizations, or those of the publisher, the editors and the reviewers. Any product that may be evaluated in this article, or claim that may be made by its manufacturer, is not guaranteed or endorsed by the publisher.

## Abbreviations

AI: artificial intelligence
AuI: augmented intelligence
CAR-T: chimeric antigen receptor T cell
ML: machine learning
NELP: natural embedding for language processing
NLP: natural language processing
SME: subject matter expert.
